# Exact Solutions of a Mathematical Model Describing Competition and Co-Existence of Different Language Speakers

**DOI:** 10.3390/e22020154

**Published:** 2020-01-28

**Authors:** Roman Cherniha, Vasyl’ Davydovych

**Affiliations:** Institute of Mathematics, National Academy of Sciences of Ukraine, 3, Tereshchenkivs’ka Street, 01004 Kyiv, Ukraine; davydovych@imath.kiev.ua

**Keywords:** reaction–diffusion system, Lie symmetry, exact solution, traveling front, community of language speakers, tanh method, 35K57, 35K58, 35B06, 35C07

## Abstract

The known three-component reaction–diffusion system modeling competition and co-existence of different language speakers is under study. A modification of this system is proposed, which is examined by the Lie symmetry method; furthermore, exact solutions in the form of traveling fronts are constructed and their properties are identified. Plots of the traveling fronts are presented and the relevant interpretation describing the language shift that has occurred in Ukraine during the Soviet times is suggested.

## 1. Introduction

It has been well known for at least 100 years that many processes arising in physics, chemistry, ecology etc. can be adequately described only by nonlinear partial differential (integro-differential, functional-differential) equations (see, e.g., an extensive discussion on this matter in Chapter 1 of [1]). During the second half of the last century, one may note also a rapidly growing number of papers devoted to applications of nonlinear partial differential equations for mathematical modeling in life sciences (see, e.g., the classical book [2], the recent monographs [3,4] and references therein).

On the other hand, the rigorous mathematical models came to social sciences and humanities only recently. In particular, papers devoted to rigorous mathematical modeling interaction of communities (populations) of different language speakers were published only during the last two decades [5,6,7,8,9,10,11]. These models are based on nonlinear differential equations of reaction–diffusion type.

We start from the nonlinear mathematical model describing interaction of three communities of language speakers, which was proposed in [10]. The model is governed by three nonlinear reaction–diffusion (RD) equations, which have the following form in the one-dimensional approximation (there are some misprints in [10], which are corrected here)

(1) $$\begin{array}{c} u_{t}=\lambda_{1}u_{xx}+a_{1}u\left(1-\frac{u}{K-(v+w)}\right)-c_{31}uw+c_{12}uv,\\ v_{t}=\lambda_{2}v_{xx}+a_{2}v\left(1-\frac{v}{K-(u+w)}\right)+\left(c_{13}+c_{31}\right)uw-\left(c_{12}u+c_{32}w\right)v,\\ w_{t}=\lambda_{3}w_{xx}+a_{3}w\left(1-\frac{w}{K-(u+v)}\right)-c_{13}uw+c_{32}vw. \end{array}$$

This model (of course, one needs to supply the relevant initial and boundary conditions) describes interaction of three communities of language speakers. Functions u(t,x) and w(t,x) describe frequencies of monolingual speakers, i.e., they speak always (or almost always) native language. Function v(t,x) stands for community of speakers, who fluently speak both languages and use each language depending on circumstances. Time derivatives $u_{t},v_{t}$ and $w_{t}$ indicate the rate of change in these frequencies, while the space-derivatives describe mobility (diffusion) in space of speakers. The second terms in each equation of (1) are some generalization of a standard logistic terms arising in many well known biological models including the famous Fisher Equation [12] and the diffusive Lotka–Volterra system (DLVS) for interacting species (see, e.g., [2,4]). The constant *K* (like in the logistic terms) means the carrying capacity of environment and defines an upper size of all three communities of speakers, i.e., it is assumed that u+v+w<K.

The language shift (a process whereby speakers of a community abandon their native language in favor of another) of some numbers of monolingual speakers to bilingual those is described by the terms $c_{31}uw$ and $c_{13}uw$. It can be noted that the language shift leads to growing the bilingual community (provided any other forces are absent).

On the other hand, the terms $c_{12}uv$ and $c_{32}vw$ describe an opposite tendency, when bilingual tends to be monolingual. It occurs, for example, in the case of the state politics leading to the lower status of one language comparing with another. The real example is the Russification in Ukraine during the Soviet period when a few million Ukrainians completely switched to the Russian language (actually, the main aim of paper [10] is to study mathematically Anglicization in Scotland). The coefficients $c_{12}$ and $c_{32}$ represent the likelihood of bilingual speakers then becoming monolingual in community *u* and *w*, respectively. Notable, the inequality $c_{12}<c_{32}$ (in particular, if $c_{12}<<c_{32}$ then one puts $c_{12}=0$) takes place if the language of community *u* is under pressure.

In paper [10], the RD system (1) was used in order to model the Anglicization process in Scotland during the 20th century. As a result, percentages of Gaelic speakers in all parts of Scotland decreased drastically. However, there is no mathematical analysis of the governing equations therein, while those were solved numerically (with the relevant boundary and initial conditions) in order to show a good correspondence between the numerical solutions and data from successive censuses.

In this paper, a modification of the RD system (1) is studied by analytical methods and a plausible interpretation of the mathematical results obtained is provided. The main results are presented in Section 2. Firstly, a modification of the system in question is proposed; secondly, Lie symmetries and a variety of exact solutions (traveling waves) are found. In Section 3, properties of the exact solutions obtained are under examination, in particular, the coefficient restrictions leading to the exact solution, which qualitatively describes the language shift occurred in Ukraine during the Soviet times, are derived. Finally, some conclusions are presented and future work is proposed in the last section.

## 2. Main Results

The RD system (1) contains fractional nonlinearities and is a very difficult task to solve analytically. Having this in mind, we propose a simpler system under biologically motivated restrictions. Our idea is to reduce fractional nonlinearities to quadratic ones. It can be noted that the fractional nonlinearities arising in (1) are a direct generalization of those introduced in the earlier work [9]. In that work, it is assumed that speakers of both languages have the common carrying capacity *K*. We think that this assumption is not well-founded because the language of a specified speaker is usually related to his/her nationality. So, one cannot claim that different nationalities have the same carrying capacities all the time. Moreover, the so-called standard model for two competing languages [7] does not use such assumption. The basic model in [7] contains the standard logistic terms arising in many biologically motivated models (see, e.g., [2,4]). Taking into account the above justification, we can replace the fractional nonlinearities by logistic terms, which also restrict unbounded growth of these communities. It means that the terms $\frac{u}{K-(v+w)},\;\frac{v}{K-(u+w)},$ and $\frac{w}{K-(v+u)}$ are replaced by $\frac{u}{K_{1}},\;\frac{v}{K_{2}}$, and $\frac{w}{K_{3}}$, respectively. As a result, we obtain the following modification of system (1)

(2) $$\begin{array}{c} u_{t}=\lambda_{1}u_{xx}+a_{1}u\left(1-\frac{u}{K_{1}}\right)-c_{31}uw+c_{12}uv,\\ v_{t}=\lambda_{2}v_{xx}+a_{2}v\left(1-\frac{v}{K_{2}}\right)+\left(c_{13}+c_{31}\right)uw-\left(c_{12}u+c_{32}w\right)v,\\ w_{t}=\lambda_{3}w_{xx}+a_{3}w\left(1-\frac{w}{K_{3}}\right)-c_{13}uw+c_{32}vw, \end{array}$$

which contains only quadratic nonlinearities. Hereafter we assume that the coefficients $\lambda_{i},\;a_{i}$ and $K_{i}$ (i=1,2,3) are positive, while all other are nonnegative (i.e., some of them can be zero).

The nonlinear RD system (2) can be simplified using the following re-scaling of the variables

$$u\to K_{1}u,\;v\to K_{2}v,\;w\to K_{3}w,\;t\to \frac{1}{a_{2}}\;t,\;x\to \sqrt{\frac{\lambda_{2}}{a_{2}}}\;x$$

and introducing new notations

$$\begin{array}{c} \alpha_{1}=\frac{c_{31}K_{3}}{a_{2}},\;\alpha_{2}=\frac{c_{12}K_{2}}{a_{2}},\;\alpha_{3}=\frac{c_{13}K_{1}}{a_{2}},\;\alpha_{4}=\frac{c_{32}K_{2}}{a_{2}},\\ \beta_{1}=\frac{a_{1}}{a_{2}},\;\beta_{3}=\frac{a_{3}}{a_{2}},\;\kappa_{1}=\frac{K_{3}}{K_{2}},\;\kappa_{2}=\frac{K_{1}}{K_{2}},\;d_{1}=\frac{\lambda_{1}}{\lambda_{2}},\;d_{3}=\frac{\lambda_{3}}{\lambda_{2}}. \end{array}$$

Thus, system (2) is reduced to the equivalent form

(3) $$\begin{array}{c} u_{t}=d_{1}u_{xx}+\beta_{1}u\left(1-u\right)-\alpha_{1}uw+\alpha_{2}uv,\\ v_{t}=v_{xx}+v\left(1-v\right)+\left(\kappa_{1}\alpha_{3}+\kappa_{2}\alpha_{1}\right)uw-\left(\kappa_{2}\alpha_{2}u+\kappa_{1}\alpha_{4}w\right)v,\\ w_{t}=d_{3}w_{xx}+\beta_{3}w\left(1-w\right)-\alpha_{3}uw+\alpha_{4}vw. \end{array}$$

Notably, system (3) with $\alpha_{1}=\alpha_{3}=0$ is a particular case of the well-known DLVS, which describes a large number of processes in biology and chemistry (see, e.g., [2,4] and references cited therein). However the above restriction is equivalent to $c_{13}=c_{31}=0$ in (2), what contradicts to the basic restrictions in the model (see interpretation of the terms $c_{31}uw$ and $c_{13}uw$). Thus, hereafter we assume that $c_{13}^{2}+c_{31}^{2}\neq 0\Leftrightarrow \alpha_{1}^{2}+\alpha_{3}^{2}\neq 0$, i.e., system (3) is not equivalent to DLVS.

It is well known that there is no general theory of integrating nonlinear partial differential equations at the present time and it is very unlikely that one will be developed soon. The most effective methods for constructing *particular exact solutions* of nonlinear differential equations of reaction–diffusion type are the classical Lie method and its various generalizations (see, e.g., the recent monographs [1,13,14] for more details). Here we apply the classical Lie method and the so-called tanh method [15,16,17].

**Theorem** **1.** *The nonlinear system (3) for any set of specified nonnegative coefficients with the additional restrictions $d_{1}d_{3}\kappa_{1}\kappa_{2}\neq 0$ and $\alpha_{1}^{2}+\alpha_{3}^{2}\neq 0$ is invariant only with respect to the time and space translations generated by Lie symmetries*

(4) $$P_{t}=\frac{\partial }{\partial t},\;P_{x}=\frac{\partial }{\partial x}.$$

The proof is based on application of the well known Lie’s algorithm to system (3) and is reduced to examination of several cases depending on values of the coefficients arising in the system. We omit here the relevant calculations. Notably, a detailed proof is presented in our recent paper [18] for a similar (but inequivalent) three-component system.

**Remark** **1.** *In contrast to the three-component DLVS, which admits some nontrivial Lie symmetries (provided its coefficients are correctly specified) [4,19], the RD system (3) possesses a poor symmetry.*

It is well known that the Lie symmetries (4) generate only two inequivalent substitutions (following the classical Sophus Lie papers, the terminology “ansatz” is often used), which reduce system (3) to the relevant systems of ordinary differential equations (ODEs). The first ansatz does not depend on the space variable *x*, hence one leads only to time-dependent solutions. Here, we are not interested in such types solutions because their realistic interpretation is questionable.

The second ansatz follows from the linear combination $P_{t}+\mu P_{x}$ of the Lie symmetries (4) and has the form

(5) u=U(ω),v=V(ω),w=W(ω),ω=x−μt,μ∈R.

Here U,V and *W* are new unknown functions. Solutions of form (5) is often called plane wave solutions (traveling waves). From the applicability point of view, the most interesting those are *traveling fronts*, i.e., solutions (5), which are bounded and nonnegative. A huge number of papers is devoted to construction of traveling fronts for nonlinear PDEs, especially for scalar reaction–diffusion (with/without convection term). Traveling fronts for such equations are presented in the monograph [20] (see also the handbook [21]).

In the case of nonlinear RD systems, the progress is rather modest. To the best of our knowledge, an essential progress is derived only in the case of DLVS. Several traveling fronts are constructed in [4,22,23,24] for the two-component DLVS and in [25,26] for the three-component DLVS.

So, our aim is to find traveling fronts for system (3). Substituting ansatz (5) into system (3), one obtains

(6) $$\begin{array}{c} d_{1}U^{\prime \prime }+\mu \;U^{\prime }+\beta_{1}U\left(1-U\right)-\alpha_{1}UW+\alpha_{2}UV=0,\\ V^{\prime \prime }+\mu \;V^{\prime }+V\left(1-V\right)+\left(\kappa_{1}\alpha_{3}+\kappa_{2}\alpha_{1}\right)UW-\left(\kappa_{2}\alpha_{2}U+\kappa_{1}\alpha_{4}W\right)V=0,\\ d_{3}W^{\prime \prime }+\mu \;W^{\prime }+\beta_{3}W\left(1-W\right)-\alpha_{3}UW+\alpha_{4}VW=0. \end{array}$$

System (6) is a three-component system of nonlinear second-order ODEs. Although this system is simpler than the original RD system (3), we can say nothing about its integrability because even the similar system obtained by reducing of the two-component DLVS has been not solved in [4,22,23,24]. In order to find particular solutions of (6), we start from the steady-state points. Obviously that steady-state points of (6) coincide with the stationary (homogenous) those of the RD system (3) and can be easily calculated by solving algebraic equations. Assuming $u_{0}v_{0}w_{0}=0$, the full list of steady-state points are as follows

(7) $$\begin{array}{c} (0,0,0),\;(0,1,0),\;(0,0,1),\;(1,0,0),\\ \left(\frac{\beta_{1}+\alpha_{2}}{\beta_{1}+\kappa_{2}\alpha_{2}^{2}},\frac{\beta_{1}\left(1-\kappa_{2}\alpha_{2}\right)}{\beta_{1}+\kappa_{2}\alpha_{2}^{2}},0\right),\;\left(0,\frac{\beta_{3}\left(1-\kappa_{1}\alpha_{4}\right)}{\beta_{3}+\kappa_{1}\alpha_{4}^{2}},\frac{\beta_{3}+\alpha_{4}}{\beta_{3}+\kappa_{1}\alpha_{4}^{2}}\right). \end{array}$$

Obviously there are also steady-state points $\left(u_{0},v_{0},w_{0}\right)$, where $u_{0}v_{0}w_{0}\neq 0$, however we prefer examine this case elsewhere. Notably, the 3rd and 4th points, like the 5th and 6th, are equivalent because the first and third equations of system (6) have the same structure. So, without loss of generality we may say that there are only four essentially different points in (7).

Typically, each traveling front possesses the following property: such a solution connects two steady-state points provided ω→±∞. We were able to identify the relevant traveling fronts in the cases listed below.

**Case 1**. $\left(U_{0},V_{0},0\right)=\left(\frac{\beta_{1}+\alpha_{2}}{\beta_{1}+\kappa_{2}\alpha_{2}^{2}},\frac{\beta_{1}\left(1-\kappa_{2}\alpha_{2}\right)}{\beta_{1}+\kappa_{2}\alpha_{2}^{2}},0\right)$ (as ω→−∞) and (0,0,1) (as ω→+∞).

**Case 2**. $\left(U_{0},V_{0},0\right)$ (as ω→−∞) and (0,0,0) (as ω→+∞).

**Case 3**. (1,1,0) (as ω→−∞) and (0,1,0) (as ω→+∞). This case occurs provided the additional restriction $\alpha_{2}=0$ takes place.

Let us consider **Case 1** and use the tanh method. To the best of our knowledge paper [15] is one of the earliest works devoted to the tanh method (there are a lot recent papers, see, e.g., [17,27] and papers cited therein). However, it can be noted that there are not many papers devoted to application of this method to nonlinear systems of PDEs. The method is essentially based at the ad hoc ansatz [15]

(8) $$u\left(t,x\right)=U\left(\omega \right)=\sum_{i=0}^{N}\gamma_{i}Y^{i},$$

where Y=tanhω. The highest power *N* should be determined by balancing the highest degree terms in *Y*, upon substitution of ansatz (8) into the equation in question. The known relation ${\left(\tanh\omega \right)}^{\prime }=1-\tanh^{2}\omega$ is essentially used when one makes balancing. Typically direct calculation show that N≤2 for the second-order PDEs. So, we obtain ansatz

$$U\left(\omega \right)=\gamma_{0}+\gamma_{1}\tanh\omega +\gamma_{2}\tanh^{2}\omega .$$

Having the correctly-specified *N*, unknown parameters $\gamma_{i}$ can be easily calculated (some of them are arbitrary constants). Of course, it often happens that N=0, therefore a trivial solution is only obtained. So, the tanh method is not applicable to a wide range of nonlinear equations. It turns out that this technique works in the case of system (6).

Thus, using ansatz (8), we may look for traveling fronts of the form

(9) $$\begin{array}{c} U=\sigma_{1}{\left(1-\tanh\omega \right)}^{n_{1}},\;V=\sigma_{2}{\left(1-\tanh\omega \right)}^{n_{2}},\;W=1-\sigma_{3}{\left(1-\tanh\omega \right)}^{n_{3}}, \end{array}$$

where $\sigma_{i}$ and $n_{i}$ (i=1,2,3) are real and natural numbers, respectively. Since the exact solution of the form (9) connects steady-state points $\left(U_{0},V_{0},0\right)$ and (0,0,1), one immediately obtains the sigma-s values

(10) $$\sigma_{1}=\frac{\beta_{1}+\alpha_{2}}{2^{n_{1}}\left(\beta_{1}+\kappa_{2}\alpha_{2}^{2}\right)},\;\sigma_{2}=\frac{\beta_{1}\left(1-\kappa_{2}\alpha_{2}\right)}{2^{n_{2}}\left(\beta_{1}+\kappa_{2}\alpha_{2}^{2}\right)},\;\sigma_{3}=\frac{1}{2^{n_{3}}}.$$

Substituting (9) into system (6) and taking into account (10), one can determine sufficient conditions for the coefficients $n_{i}$ when the traveling fronts can be found explicitly.

Omitting the relevant calculations, we only present the result. So, system (3) has the exact solution

(11) $$\begin{array}{c} u=\frac{6d_{1}}{\beta_{1}}{\left(1-\tanh(x-\mu t)\right)}^{2},\\ v=\frac{24d_{1}-\beta_{1}}{2\alpha_{2}}\left(1-\tanh(x-\mu t)\right),\\ w=\frac{1}{2}+\frac{1}{2}\tanh\left(x-\mu t\right) \end{array}$$

provided its coefficients satisfy the restrictions:

(12) $$\begin{array}{c} \alpha_{1}=16d_{1}-4\mu +\beta_{1},\;\alpha_{3}=\frac{d_{3}\beta_{1}}{3d_{1}},\;\kappa_{1}=\frac{5-2\mu }{\alpha_{4}},\;\kappa_{2}=\frac{\beta_{1}\left(\alpha_{2}+\beta_{1}-24d_{1}\right)}{24d_{1}\alpha_{2}^{2}},\\ \beta_{1}=\frac{2\alpha_{2}^{2}\left(\alpha_{4}+\left(2\mu -5\right)d_{3}\right)}{\left(10d_{1}-\mu +2\alpha_{2}\right)\alpha_{4}}+24d_{1}-\alpha_{2},\;\beta_{3}=\frac{2\left(2d_{3}-\mu \right)\alpha_{2}+\left(\beta_{1}-24d_{1}\right)\alpha_{4}}{\alpha_{2}}. \end{array}$$

The second exact solution

(13) $$\begin{array}{c} u=\frac{\beta_{1}+\alpha_{2}}{4\left(\beta_{1}+\kappa_{2}\alpha_{2}^{2}\right)}{\left(1-\tanh(x-10t)\right)}^{2},\\ v=\frac{\beta_{1}\left(1-\kappa_{2}\alpha_{2}\right)}{4\left(\beta_{1}+\kappa_{2}\alpha_{2}^{2}\right)}{\left(1-\tanh(x-10t)\right)}^{2},\\ w=1-\frac{1}{4}{\left(1-\tanh(x-10t)\right)}^{2}, \end{array}$$

was constructed provided the coefficients of system (3) satisfy the restrictions:

(14) $$\begin{array}{c} d_{1}=1,\;d_{3}=1,\;\alpha_{1}=\beta_{1}-24,\\ \kappa_{1}=\frac{24\alpha_{2}\kappa_{2}+23\beta_{1}-\left(\beta_{1}-24+24\alpha_{2}\right)\beta_{1}\kappa_{2}}{\left(\alpha_{3}-\alpha_{4}\right)\beta_{1}+\left(\alpha_{3}+\alpha_{4}\beta_{1}\kappa_{2}\right)\alpha_{2}},\;\beta_{3}=\frac{\left(\alpha_{3}-\alpha_{4}-24\right)\beta_{1}-24\kappa_{2}\alpha_{2}^{2}+\left(\alpha_{3}+\alpha_{4}\beta_{1}\kappa_{2}\right)\alpha_{2}}{\beta_{1}+\kappa_{2}\alpha_{2}^{2}}. \end{array}$$

It is easily seen that the traveling front (11) is more general than (13), since its velocity μ is not fixed.

In **Case 2**, taking into account the corresponding steady-state points, we are looking for the traveling fronts in the form

(15) $$\begin{array}{c} U=\frac{\beta_{1}+\alpha_{2}}{2^{n_{1}}\left(\beta_{1}+\kappa_{2}\alpha_{2}^{2}\right)}{\left(1-\tanh\omega \right)}^{n_{1}},\\ V=\frac{\beta_{1}\left(1-\kappa_{2}\alpha_{2}\right)}{2^{n_{2}}\left(\beta_{1}+\kappa_{2}\alpha_{2}^{2}\right)}{\left(1-\tanh\omega \right)}^{n_{2}},\\ W=\sigma \left(1-\tanh^{2}\omega \right), \end{array}$$

where σ is an unknown positive constant. Substituting (15) into system (6) and making the corresponding calculations, we arrive at the exact solution

(16) $$\begin{array}{c} u=\frac{17-16d_{1}+\alpha_{2}}{68-64d_{1}+4\kappa_{2}\alpha_{2}^{2}}{\left(1-\tanh\left(x-\frac{17}{4}\;t\right)\right)}^{2},\\ v=\frac{\left(17-16d_{1}\right)\left(1-\kappa_{2}\alpha_{2}\right)}{68-64d_{1}+4\kappa_{2}\alpha_{2}^{2}}{\left(1-\tanh\left(x-\frac{17}{4}\;t\right)\right)}^{2},\\ w=\frac{17-40d_{1}}{4\alpha_{1}}\left(1-\tanh^{2}\left(x-\frac{17}{4}\;t\right)\right). \end{array}$$

The traveling front (16) satisfies system (3) if the coefficient restrictions

(17) $$\begin{array}{c} \beta_{1}=17-16d_{1},\;\beta_{3}=\frac{17-8d_{3}}{2},\;\alpha_{4}=\frac{16d_{1}\left(17-\alpha_{3}\right)+\left(17+\alpha_{2}\right)\alpha_{3}-17\left(17+\kappa_{2}\alpha_{2}^{2}\right)}{\left(17-16d_{1}\right)\left(1-\kappa_{2}\alpha_{2}\right)},\\ d_{3}=\frac{17}{8}-\frac{17\alpha_{1}}{12\alpha_{1}+80d_{1}-34},\;\kappa_{1}=\frac{\alpha_{1}}{17(17-40d_{1})}\frac{391-368d_{1}-\left(289-952d_{1}+640d_{1}^{2}+408\alpha_{2}-408d_{1}\alpha_{2}\right)\kappa_{2}}{17-16d_{1}+\kappa_{2}\alpha_{2}^{2}} \end{array}$$

are satisfied.

Finally, in **Case 3**, the exact solutions of system (6) were prescribed to have the form

$$\begin{array}{c} U=\frac{1}{2^{n_{1}}}{\left(1-\tanh\omega \right)}^{n_{1}},\;V=1+\sigma_{2}\left(1-\tanh^{2}\omega \right),\;W=\sigma_{3}\left(1-\tanh^{2}\omega \right). \end{array}$$

After the relevant calculations, the traveling front

(18) $$\begin{array}{c} u=\frac{1}{4}{\left(1-\tanh\left(x-\frac{\alpha_{3}}{4}\;t\right)\right)}^{2},\\ v=1+\frac{24-\alpha_{3}}{2(\alpha_{3}-8)}\left(1-\tanh^{2}\left(x-\frac{\alpha_{3}}{4}\;t\right)\right),\\ w=\frac{\alpha_{3}-40d_{1}}{4\alpha_{1}}\left(1-\tanh^{2}\left(x-\frac{\alpha_{3}}{4}\;t\right)\right), \end{array}$$

of the nonlinear system (3) was derived provided the coefficient restrictions

(19) $$\begin{array}{c} \alpha_{2}=0,\;\beta_{1}=-16d_{1}+\alpha_{3},\;\beta_{3}=\frac{2\alpha_{1}\alpha_{3}\left[\alpha_{3}-2\left(4+\alpha_{4}\right)\right]}{\left(\alpha_{3}-8\right)\left(40d_{1}+6\alpha_{1}-\alpha_{3}\right)},\;d_{3}=\frac{\alpha_{3}-2\alpha_{4}-2\beta_{3}}{8},\\ \kappa_{1}=\frac{\alpha_{1}\left(\alpha_{3}-24\right)\left(\alpha_{3}-6\right)}{\alpha_{4}\left(\alpha_{3}-8\right)\left(\alpha_{3}-40d_{1}\right)},\;\kappa_{2}=\frac{\alpha_{3}\left(6-\alpha_{3}+2\alpha_{4}\right)}{\alpha_{1}\left(\alpha_{3}-6\right)}\kappa_{1}, \end{array}$$

take place.

**Remark** **2.** *In Cases 1–3 there exist such sets of the positive parameters (excepting $\alpha_{2}=0$ in **Case 3**)*

$$d_{1},\;d_{3},\;\alpha_{i},\;\beta_{1},\;\beta_{2},\;\kappa_{1},\;\kappa_{2},$$

*satisfying the restrictions (12), (14), (17) and (19) that three components of the exact solutions (11), (13), (16) and (18), respectively, are positive. Thus, all the solutions obtained are indeed traveling fronts.*

**Remark** **3.** *It can be easily checked that all the solutions derived above satisfy the zero Neumann conditions at x→±∈. In the case of a bounded domain (A,B), one obtains at the boundaries $u_{x}\approx 0,\;v_{x}\approx 0$ and $w_{x}\approx 0$ provided |A| and |B| are sufficiently large. Such boundary conditions (they often called no flux conditions) are typical requirements in many real-world models and, for instance, were used in [10].*

## 3. Interpretation of Traveling Fronts

In this section, we study in detail exact solution (11). First of all, we answer the question: When do positive coefficients $d_{1},\;d_{3},\;\alpha_{2},\;\alpha_{4}$ and μ lead automatically to positive values of $\alpha_{1},\;\alpha_{3},\;\beta_{1},\;\beta_{3},\;\kappa_{1}$ and $\kappa_{2}$ in formulae (12)? It turns out that some additional restrictions are needed. The structure of such restrictions essentially depends on the sign of the parameter μ, i.e., on the traveling front direction. Thus, one needs to examine separately two cases: **(i)** μ>0 and **(ii)** μ<0.

In Case **(i)**, one immediately obtains $0<\mu <\frac{5}{2}$ (see the formula for $\kappa_{1}$ in (12)). For a simplicity, we assume additionally $\alpha_{2}=\alpha_{4}\equiv \alpha$ and introduce the notations

$$F\equiv 10d_{1}-\mu +2\alpha ,\;G\equiv 2\mu d_{3}-5d_{3}+\alpha .$$

Substituting these notations into (12), we arrive at the system of the inequalities:

(20) $$\begin{array}{c} FG>0,\;\alpha_{1}=40d_{1}-4\mu -\alpha \left(1-2\;\frac{G}{F}\right)>0,\\ \beta_{1}=24d_{1}-\alpha \left(1-2\;\frac{G}{F}\right)>0,\;\beta_{3}=4d_{3}-2\mu -\alpha \left(1-2\;\frac{G}{F}\right)>0. \end{array}$$

Since all the component of (11) should be nonnegative (we remind the reader that each component means a frequency of the community speakers), the inequality $\beta_{1}<24d_{1}$ takes place, which follows from V≥0. Thus, the restriction $\frac{G}{F}<\frac{1}{2}$ is obtained. It can be also noted that F>0 and G>0 (the case F<0 and G<0 leads to a contradiction).

In order to satisfy all the inequalities in (20), we set

$$G=\varepsilon \Leftrightarrow \alpha =\left(5-2\mu \right)d_{3}+\varepsilon ,$$

where ε>0 is a sufficiently small parameter. Now the 4th inequality in (20) is reduced to the form:

(21) $$d_{3}\geq \frac{2\mu +\varepsilon }{2\mu -1},$$

hence $\mu >\frac{1}{2}.$ The 2nd and 3rd those are satisfied provided

(22) $$40d_{1}>4\mu +5d_{3}-2\mu d_{3}+\varepsilon ,\;24d_{1}>5d_{3}-2\mu d_{3}+\varepsilon .$$

Now one realizes that the following algorithm guarantees the positivity of all the coefficients in (12). Firstly, we fix any μ from the interval $\left(\frac{1}{2},\frac{5}{2}\right)$ and a small ε, say ε<1. Secondly, we take any $d_{3}$ satisfying (21) and calculate $\alpha =\left(5-2\mu \right)d_{3}+\varepsilon .$ Finally, we choose a sufficiently large $d_{1}>0$ in order to satisfy inequalities (22).

**Remark** **4.** *In the case $\alpha_{2}=\alpha_{4}\equiv \alpha$ and $d_{1}=d_{3}\equiv d$, the above algorithm is simplified to the identification of the restrictions $d\geq \frac{2\mu +\varepsilon }{2\mu -1}$ and α=(5−2μ)d+ε, where $\varepsilon >0,\mu \in \left(\frac{1}{2},\frac{5}{2}\right)$.*

Case **(ii)** is essentially simpler. In fact, one immediately obtains $\alpha_{1}>0$ and $\kappa_{1}>0$ in (12). Assuming additionally that $\alpha_{2}=24d_{1}$ and solving the inequalities $\beta_{1}>0$ and $\beta_{3}>0$ (see (12)), we obtain the restrictions

$$\begin{array}{c} \alpha_{2}=24d_{1},\;d_{3}<1,\;\mu <\frac{d_{3}}{2(d_{3}-1)},\\ \left(5-2\mu \right)d_{3}<\alpha_{4}<\frac{2}{10d_{1}-\mu }\left(\mu^{2}+2\left(24d_{1}d_{3}-d_{3}-29d_{1}\right)\mu -4d_{1}d_{3}\right), \end{array}$$

which guarantee the positivity of all the coefficients in (12).

**Figure 1:**
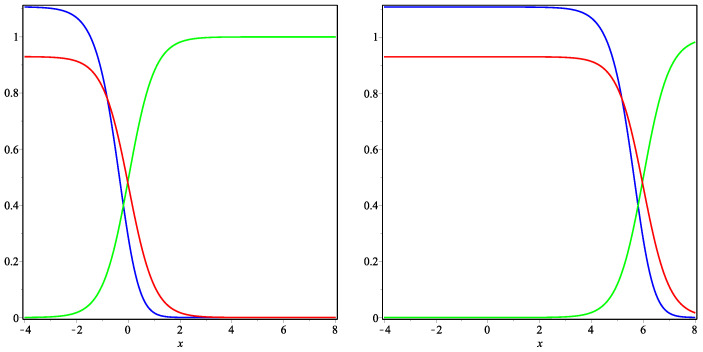
Traveling fronts (11). Curves represent the functions $u(t_{0},x)$ (blue represents the Russian speakers), $v(t_{0},x)$ (red represents the bilingual speakers) and $w(t_{0},x)$ (green represents the Ukrainian speakers) for the fixed time $t_{0}=0.01$ (**left**) and $t_{0}=4$ (**right**) and the parameters $\mu =\frac{3}{2},\;d_{1}=d_{3}=2,\;\alpha_{2}=\alpha_{4}=5$ (other parameters are calculated by formulae (12)).

**Figure 2:**
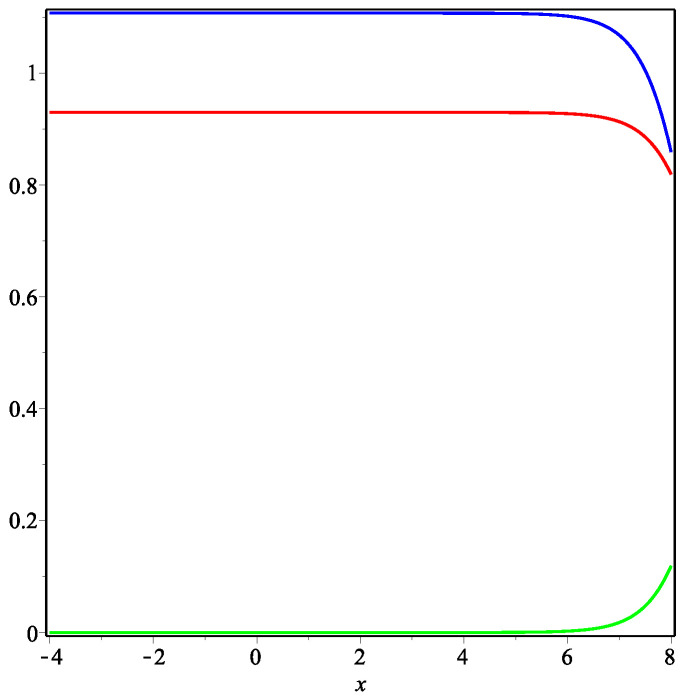
Traveling fronts (11). Curves represent the functions $u(t_{0},x)$ (blue), $v(t_{0},x)$ (red) and $w(t_{0},x)$ (green) for the fixed time $t_{0}=6$ and the parameters $\mu =\frac{3}{2},\;d_{1}=d_{3}=2,\;\alpha_{2}=\alpha_{4}=5$ (other parameters are calculated by formulae (12)).

**Figure 3:**
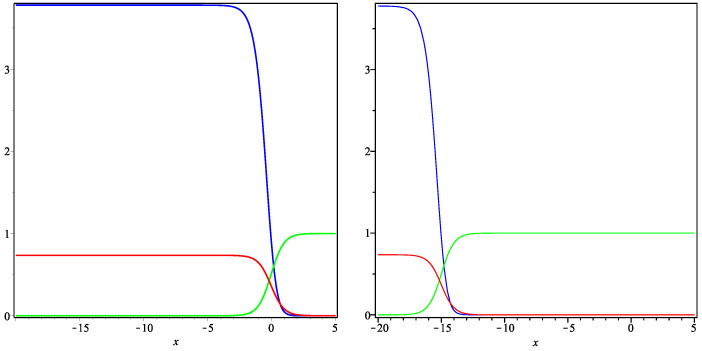
Traveling fronts (11). Curves represent the functions $u(t_{0},x)$ (blue), $v(t_{0},x)$ (red) and $w(t_{0},x)$ (green) for the fixed time $t_{0}=0.01$ (**left**) and $t_{0}=3$ (**right**) and the parameters $\mu =-5,\;d_{1}=d_{3}=\frac{1}{2},\;\alpha_{2}=\alpha_{4}=12$ (other parameters are calculated by formulae (12)).

Thus, we can use the formulae derived above in order to construct examples of traveling fronts to plot the relevant curves (using the package Maple) and to present their plausible interpretation. Figure 1, Figure 2 and Figure 3 represent the exact solution (11) in Case **(i)** μ>0 (Figure 1 and Figure 2) and Case **(ii)** μ<0 (Figure 3). All the curves satisfy the natural requirement of positivity at the given space intervals.

In Figure 1 and Figure 2, three traveling fronts are moving to the right along the OX axes as it is predicted in Case **(i)**. If we assume that the blue and green curves represent the communities of Russian language speakers and Ukrainian language speakers, while the red curve describes the frequency of bilingual speakers, then the real language shift occurred in Ukraine during the Soviet period (from the end of the Second WW till the USSR collapse) is qualitatively described by these curves. In fact, the language situation in Ukraine can be approximated by the 1D model because the communities of different language speakers varies very essentially from east to west (not so much from north to south).

So, taking the point (x=−4.0) as the eastern end and the point (x=8.0) as the western end, one realizes that the above curves at the time moment t=0.01 (see the curves in the left part of the figure) reflects the situation in the end of the Second WW (the borders of the modern Ukraine were formed in that time). The frequency of Russian language speakers (blue curve) was very high in the eastern part (see the interval x∈[−4,−2]), while an opposite situation was in the western part (interval x∈[6,8]), in which Ukrainian language dominated (actually the Russian language was unknown therein). In the central part of Ukraine (interval x∈[−2,6]), the linguistic situation was more complicated and this is shown in Figure 1 (left plot). However, one may say that Ukrainian language speakers (green curve) formed the main part of inhibitors of the Central Ukraine and the frequency of using this language decreased in the eastern direction. Finally, the community of bilingual speakers (red curve) was concentrated mostly in the east part after the end of the Second WW.

The time moment t=4.0 (see the curves in the right part of the Figure 1) reflects the situation in the end of Soviet times, i.e., in the beginning of 1990s. In that time, the community of Russian language speakers (blue curve) dominated in the east and central part of Ukraine (interval x∈[−4,6]), the community of bilingual speakers (red curve) was also strong in these parts. However, the frequency of using Ukrainian language was very low and one may say about a rapid extinction of this community. In that time, Ukrainian language dominated only in the western part of Ukraine, while there was also a part of the Central Ukraine, in which the frequencies of using both languages was in some equilibrium (interval x∈[4,6]).

Traveling fronts presented in Figure 2 model the situation under the assumption that the USSR could exist 20–30 years longer doing the same language politics, which was in favor of Russian language. Of course, one can expect the almost complete extinction of Ukrainian language speakers as it is shown (see green curve), however existence of a large community of bilingual speakers (red curve) seams to be not plausible. In fact, there is no any reason to study a ‘dead’ language. So, we believe that the red curve does not describe adequately the frequency of using both languages for large values of time.

In Figure 3, the exact solution (11) is pictured in Case **(ii)** μ<0, so that the traveling fronts are moving to the left. As a result, the relevant interpretation is different. In fact, the time evolution leads to extinction of two communities, while only one monolingual community is the winner of this language competition.

Finally, it should be pointed out that the exact solutions of the form (11) used above for interpretation of the language shift occurred in Ukraine during the Soviet times do not express exact numbers of speakers, however these solutions describe qualitatively the real linguistic situation. In order to get accurate quantitative results, one needs to calculate correct coefficients in the RD system (2) using census data in the former USSR. This is another nontrivial problem, which will be treated elsewhere.

## 4. Conclusions

In this work, the known three-component reaction–diffusion system modeling the competition and co-existence of two different language speakers [10] was a starting point. Such competition leading to a language shift occurs in many countries (territories) and Ukraine is a typical example. A modification of this system is proposed (see system (3)), which was examined by the Lie symmetry method. It was established that the system in question is invariant only w.r.t. the Lie operators of the time and space translations provided its coefficient satisfy natural restrictions. Furthermore, exact solutions in the form of traveling fronts are constructed using the tanh function technique. As a result, four exact solutions in explicit form were found for the first time. One of them (see formulae (11)) was studied in detail in order to identify its properties. Having this done, plots of the traveling fronts were drown and the relevant interpretation describing the language shift that occurred in Ukraine during the Soviet times was suggested.

We are going to continue this work. In particular, some extension of the model is needed in order to take into account possible changes in language politics introduced by the government.

Finally, it should be noted that a three-component model for describing the spread of an initially localized population of farmers into a region occupied by hunter-gatherers was introduced in [28] (see also the recent paper [18], in which traveling fronts are constructed). It can be shown that the farmer–hunter-gatherers model can be derived from the RD system (2) as a particular case.
